# Mapping from spatial meaning: bridging Hñahñu (Otomi) ecological knowledge and geo-information tools

**DOI:** 10.1186/s13002-019-0329-9

**Published:** 2019-10-11

**Authors:** José María León Villalobos, Verónica Vázquez García, Enrique Ojeda Trejo, Michael K. McCall, Juan Hernández Hernández, Gaurav Sinha

**Affiliations:** 1 Centro de Investigación en Ciencias de Información Geoespacial A.C.(CentroGeo), Contoy 137, Col. Lomas de Padierna, Delegación. Tlalpan, C.P. 14240 Cuidad de México, Mexico; 2 Posgrado en Estudios del Desarrollo Rural, Colegio de Postgraduados, Campus Montecillo, Carretera México-Texcoco Km. 36.5, C.P, 56230 Texcoco, Estado de Mexico Mexico; 3 Posgrado en Edafología, Colegio de Postgraduados, Campus Montecillo, Carretera México-Texcoco Km. 36.5, C.P, 56230 Texcoco, Estado de Mexico Mexico; 4 Centro de Investigaciones en Geografía Ambiental (CIGA), Universidad Nacional Autónoma de México (UNAM), Antigua Carretera a Pátzcuaro No. 8701. Col. Ex-Hacienda de San José de la Huerta, C.P, 58190 Morelia, Michoacan Mexico; 5 Asociación de Usuarios Alto Tunititlán A.C., Av. Coyoacán No. 4, Col. Huitexcalco de Morelos, C.P. 42753, Chilcuautla, Hidalgo Mexico; 6 Department of Geography, Ohio University, Athens, OH 45701 USA

**Keywords:** Local taxonomy, Landscape categories, Spatial language, Participatory mapping

## Abstract

**Background:**

Hñahñu (Otomi) farmers organize their experiences and ecological learning into a farmland system designed to grow food in areas of scarce water and low soil fertility. The purpose of this paper is to examine Hñahñu concepts and categories pertaining to the farming landscape and the ecological foundations underlying the system, its management implications, and categorial organization in Huitexcalco de Morelos, Mezquital Valley, Mexico.

**Methods:**

Native terms and their links to landscape were recorded and discussed in various workshops. Open interviews and field trips with local experts were used to explain soil and water management practices that allow Hñahñu farmers to maintain sustained yields throughout the year. We then used participatory mapping in order to explore the semantic relations of the terms with the space and its validity in the productive landscape.

**Results:**

We elicited 7 Hñahñu language terms related to landforms, 4 related to land use categories, and 17 related to their constituent components organized in two hierarchical levels. We found that *mothe* as a term of land usage was followed by *mothee*, *ñut’athee*, *gadñhe*, or *muiñhe*; these primarily refer to the topographic position of the parcel and the form of access to water for irrigation. Stone barriers and earth channels represent the functional structures that are most commonly used by Hñahñu farmers to retain soil and water. In the participatory mapping results, *mothe muiñhe* displayed a robust spatial link with the gullies. Identifying other landscape categories required a substantial understanding both of management practices of soil and water and forms of organization.

**Conclusions:**

This study revealed a complex system of knowledge that contributes to the continued proper management of the local landscape. The terms and their elicited meanings are key to understand the ways in which Hñahñu farmers conceptualize and relate the reality of their landscape and its cultural meanings. Scale and perception were found to have a determining role in defining their taxonomic organization, semantic structure, and relations in space.

## Background

Traditional knowledge systems reflect the cognitive experiences of human groups worldwide [1]. Not only do they synthesize the diverse learning, concepts, and customs attributed to interactions between local farmers and their landscapes, but they also express the different ways in which people structure and organize, in categories and hierarchies, their cognitive experiences [2–4]. This historical engagement of local farmers with their cultural and natural environment is exemplified in how they codify, process, categorize, and attach significance to their experiences [2, 5].

Local farming systems are a good example of how local people organize and structure their learning and knowledge. Research has shown that farmers have a broad knowledge of plant and soil interactions and climate cycles which they effectively integrate into complex land sustainable management patterns and practices [6–8]. By manipulating soil and rainwater, farmers directly improve soil fertility and moderate natural climate variations of the local environment, thus sustaining production yields [9, 10]. Furthermore, findings on three continents (Africa, America, and Asia) suggest close associations between local and scientific systems [11–13]. Local categories can be found in the most commonly used scientific classification schemes [14, 15].

Recent research on farming landscape patterns has revealed that native farmers have multiple and varied ways of interacting with their vertical landscape and physical environment by classifying them into hierarchies and categories of land use [16–19]. Salient biotic and abiotic factors including elevation, relief, and aspect as well as associations or locations of organisms are commonly utilized to distinguish the categories [20]. While landscape categories are indeed reflected in native languages, native terms have rarely been used as an information source to examine categorizations of landscape. It is only through the recent intersection of anthropology, linguistics, and geography that researchers have started to examine the ways in which local people categorize landscape [21]. Native language is key to elucidate how landscape is conceptualized in cognitive entities and how the organization of such entities occurs in local classification systems represented in thought [22–25].

This paper focuses on the Hñahñu (Otomi) farmers of the Mezquital Valley. Their farming knowledge system classifies land types according to their productive value. We use a transdisciplinary approach that involves landscape recognition and ethnosciencies in order to document and explain how Hñahñu farmers maintain several species and crop varieties by actively managing soil and rainwater [26]. We show how a participatory mapping exercise helped to relate the elicited terms with concrete spatial locations in the local landscape [27, 28].

## Methods

**Fig. 1:**
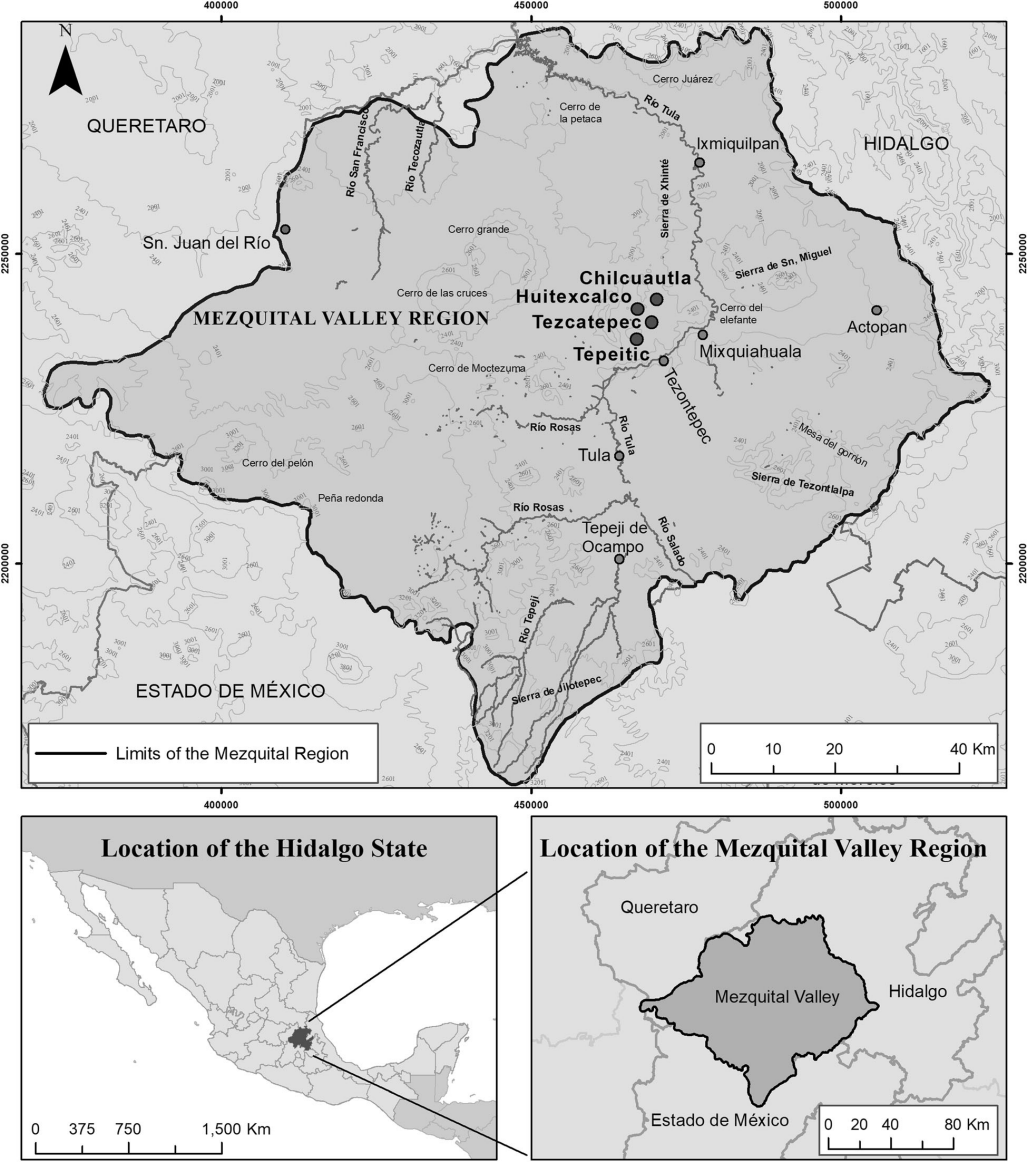
Location of Huitexcalco de Morelos in the Mezquital Valley of Hidalgo, Mexico

Huitexcalco de Morelos, situated in the Mezquital Valley, is inhabited by around 1560 people, most of them (86%) speakers of the Hñahñu language (Fig. 1). Over the years, local farmers have been able to integrate conservation practices into an agricultural system characterized by scarce natural resources, especially water [29]. During the late Spanish colonial period (1750–1821), Spaniards appropriated the best lands for cropping while Hñahñus were pushed to meager arable lands [30]. Hñahñu farmers combined soil and water conservation techniques with efficient crop selection. They raised stone walls in river beds, gully, and hillsides in order to retain runoff water with sediments, thus creating fertile terraces on which they could grow maize, beans, squash, and agave [31].

The agrarian reform following the Mexican Revolution of 1910 allowed for the Hñahñus to gain access to more fertile lands and better-quality pastures [32]. During the second part of the twentieth century, price decline of agricultural products, coupled with peasant migration to the USA, resulted in the progressive abandonment of agriculture [33, 34]. Nevertheless, local agriculture was maintained in certain farmland parcels partly due to subsidized government programs. Currently, the Hñahñus of Huitexcalco de Morelos maintain more than 1500 ha of agricultural lands. Each farmland parcel is classified by Hñahñus using native terms in order to emphasize differences in farming practices and land productivity.

### Conceptualization of the Hñahñu agricultural system

The collection of Hñahñu terms took place in workshops held between August 2015 and January 2016. Two workshops with a total duration of 8 h were conducted in order to elicit existing Hñahñu terms linked to agriculture, using as a starting point a vocabulary list suggested by Granados et al. [35]. A total of 13 people took part in the workshops; they were selected due to their long-term farming experience and command of Hñahñu language. Five of them were elderly (70–80 years old). Following the method used by Wellen and Sieber [36], participants wrote the terms on cards and the meanings in both Hñahñu and Spanish were further discussed until consensus was finally reached.

The research included three field trips whose purpose was to identify land types that were mentioned in the workshops. An average of 10 people attended each field trip. Using an open interview guide, information on irrigation strategies, soil and water conservation practices, soil types, and crop varieties was also collected. This activity was originally designed to improve our conceptual understanding of the terms; however, it ended up contributing to organizing the information in two hierarchical levels, with terms used to describe relief (mountains, slopes, hills and plains) in the first level, and land quality in the second.

### Mapping of the Hñahñu farmland system

In order to determine whether the Hñahñu farming system categories share spatial and semantic domains, a participatory mapping exercise was conducted. Between February and March 2016, a 1:25,000 satellite image was used with the boundaries of the previously digitized farming parcels; participants were able to map the location and distribution of two farmland types: *mothe muiñhe* and *mothe ñut’athee*. They initially identified parcels which could be classified as one of these two types, and they then compared these with the adjacent parcels. An important feature in the recognition of *mothe muihñes* is that these farming parcels were found to be situated in the mouth of a gully. In contrast, in the case of *mothe ñut’athee*, it was observed that all parcels involved channeling water from ephemeral watercourses.

However, in the case where flood water rejoins the watercourse, the farmland parcel is then designated as a variant of *mothe ñut’athee*: *mothe gadñhe*. Finally, the remaining parcels are designated as *mothe mothee*. Any farmland parcel that was classified with more than one category retained the name of the dominant one (that is, encompassing over 50% of surface of the parcel).

The match between agricultural categories represented on the map and concrete locations was examined through fieldtrips to three randomly selected farmlands. Visits to some parcels of the *mothe gadñhe* type (which were difficult to identify) were also included. After agreeing upon their final boundaries, polygons of farmland categories were digitized and depicted on a map that was then presented to participants on a computer screen by using Google Earth. The final map was delivered to the community in several printed formats and different perspectives for their own archives.

## Results

### Taxonomic organization of Hñahñu knowledge

The Hñahñu farmland of Huitexcalco is classified under a two-level hierarchical scheme. The first level includes four categories of landforms: *Ya t’oho* (mountains), *bogats’i* (hillsides), *bobatha* (plains), and *bodants’i* (low hills). In turn, *Bogats’i* and *bodants’i* are divided according to their topographic position in the hills: *donts’i* (upper part), *ngats’i* (middle part), and *ngat’i* (lower part). Based on the topographic position of farmland parcels, Hñahñus can forecast their productivity through association with slope steepness, access to sources of water, and soil quality.

**Fig. 2:**
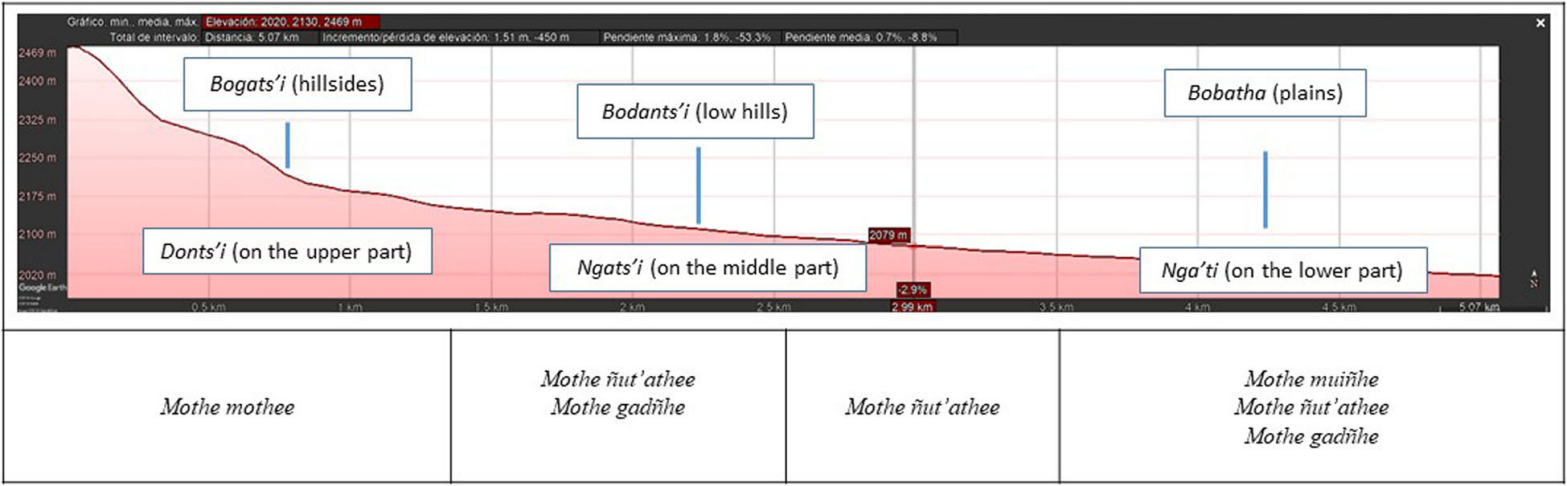
Elevation profile of Huitexcalco de Morelos, Mezquital Valley of Hidalgo, Mexico, showing the relationship between categories of landforms and farmland types in Hñahñu language

The second level entails different categories of farmland. Hñahñu farmers classify their parcels on the basis of their topographic position within the landscape, as well as the manner in which water is provided for irrigation. Interestingly, categories are named using the word *mothe*, followed by one of these words, *mothee*, *ñut’athee*, *gadñhe*, or *muiñhe*. Each term denotes the strategy adopted for collecting and diverting water to parcels. Figure 2 depicts how farmland types pertain to landforms.

### Types of landforms in Hñahñu language

Hñahñus distinguish at least four categories of landforms: *Ya t’oho* (mountains), *bogats’i* (hillsides), *bodants’i* (low hills), and *bobatha* (plains). They are also capable of identifying significant variations of slope and soil depth in each of the categories. This helps them determine land productivity and the consequent farmland practices that need to be applied.

**Fig. 3:**
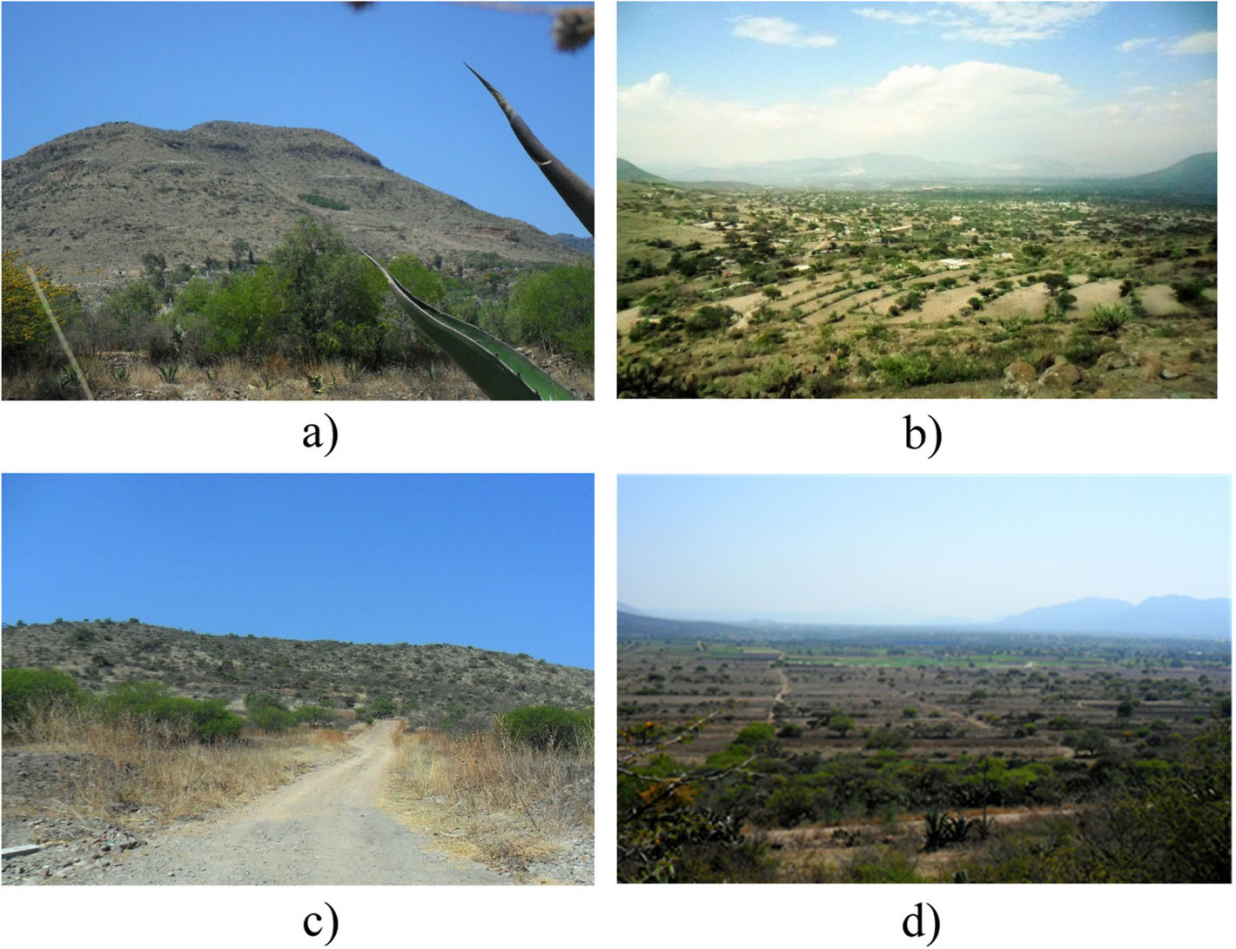
Landforms in the landscape of Huitexcalco de Morelos, Mezquital Valley, Mexico. **a** *Ya t’oho*: hills of Cerro de la Cruz. **b** *Bogats’i*: hillsides of Cerro de la Cruz. **c** *Bodants’i*: low hills near the town boundaries. **d** *Bobatha*: plains surrounding the town

The Hñahñu term *ya t’oho* signifies the most prominent topographical attributes of Huitexcalco, primarily mountains and hills (Table 1; Fig. 3a). The slope and shallow soils of low fertility of *ya t’oho* prevent crop growing. Hñahñus use these lands to collect fruits, vegetables, flowers, mushrooms, fibers, and insects. This suggests that *ya t’oho* are valued more for their ecological functions rather than their potential for arable farming.

**Table 1:** Hñahñu topographical terms and their Spanish equivalents

Hñahñu term	Pronunciation	Spanish/English equivalent
*Ya t’oho*	Ya’t’o̱ho̱a	“Cerros” (hills) and/or “montañas” (mountains)
*Bogats’i*	Bǒ’kä́ts’i	“Laderas” (hillsides) or “pie de monte” (foothill)
*Bodants’i*	Bǒ’dä́nts’i	“Lomas” or “lomeríos” (low hills)
*Bobatha*	Bǒ’bǎtha	“Planicies” (plains)
*Donts’i*	Dónt’i	“En la parte alta” (on the upper part)
*Ngats’i*	Nkä́ts’i	“En la parte media” (on the middle part)
*Nga’ti*	Ngá’ti, Nga’ti	“En la parte baja” (on the lower part)

Source: local experts from Huitexcalco de Morelos, Mezquital Valley, Mexico

*Bogats’i* is the corresponding term for the Spanish term “laderas” (hillsides) or “pie de monte” (foothill). Since slope in *bogats’i* tends to vary greatly, Hñahñus make use of the terms *donts’i*, *ngats’i*, and *ngat’i* in order to distinguish the upper, middle, and lower parts of the slopes (Table 1). *Donts’i* are stony and shallow soils, whereas *ngats’i* and *nga’ti* occurring in gentler slopes (< 5%) make up soils that are deep enough to be able to support various crops. Gullies here tend to be narrower (below 4 m), thereby making it easier to retain and divert water flow into farmland parcels during the rainy season.

The term *Bodants’i* corresponds to the Spanish term “lomas” or “lomeríos” (low hills; Table 1; Fig. 3c); Hñahñus view this as a transitional landform between hillsides and plains. Similarly to the previous category (*bogats’i*), Hñahñus use the terms *donts’i*, *ngats’i*, and *nga’ti* in order to distinguish variations in slope*.* Since flat surfaces tend to favor soil deposition, both *ngats’i* and *nga’ti* have deeper and more fertile soils, particularly those near gullies which accumulate rich sediments arriving from upstream. In addition, on *nga’ti* lands, water retention and diversion to farmland parcels need less effort because the gullies occurring on gentler slopes (5% on average) are generally shallower.

For Hñahñu farmers, *Bobatha* (equivalent to “planicies” in Spanish, meaning plains) are the most highly valued for cropping, allowing up to two harvests per year (Table 1; Fig. 3d). Both have deep soils (up to 70 cm) and flat surfaces that facilitate water runoff to arable parcels. As Table 1 shows, *bobatha* are the most productive parcels of the Hñahñu farmland system.

### Farmland categories for Hñahñus

Hñahñus of Huitexcalco use the term *mothe* in order to identify and designate various farmland parcels. Workshop participants stated that this term is made up by two words: *mo* (“retiene o capta” [retains]) and *the* (“agua” [water]). *Mothe* is the most eloquent description for a land parcel that collects or retains water. This term is combined with other words *muiñhe*, *ñut’athee*, and *mothee* in order to depict how and where in the landscape water is collected or retained for irrigation purposes (Table 2). Although the parcel’s position in the landscape and the practices used for collecting/retaining water are the primary criteria utilized by Hñahñus in order to classify their farmlands parcels, other equally important criteria include slope and soil depth.

**Table 2:** Hñahñu terms and their Spanish equivalents used to designate different farmland types and constitutive components of farmland parcels in Huitexcalco de Morelos, Mezquital Valley, Mexico

Hñahñu term	Pronunciation	Spanish/English meanings or equivalents
*Mothe muiñhe*	Móthe mú̱ñhe	Parcela que capta/retiene el agua en el lecho/panza del arroyo (farmland parcel that collects/retains water at the mouth of the gully)
*Mothe ñut’athee*	Móthe ‘ñut’déhe	Parcela que capta/retiene el agua que le entra o le llega del arroyo (farmland parcel that collects/retains incoming water from gullies or runoff)
*Mothe gadñhe*	Móthe gádñhe	Parcela que capta/retiene el agua en la ribera del arroyo y donde el agua regresa al cauce (farmland parcel that collects/retains water on the banks of gullies and from which water returns to the streambed
*Mothe mothee*	Móthe móthe	Parcela en donde se capta/retiene el agua que proviene únicamente de la lluvia (farmland parcel that collects/retains water only from rainfall)
*Jodo*	Jodo	Pared de piedra acomodada (stone wall)
*Ndosthee ga do*	Ndósthee	Barrera de piedra (stone barrier)
*Ñuthee njushai*	‘Ñúthe ‘ñǔshai	Camino o canal del agua escarbado en tierra (waterway or channel dug into the ground)
*Sokdehe*	Sókděhe	Vertedero (spillway)
*‘Bohai*	Bo̱hai	Suelo oscuro (dark soil)
*Picahai*	Pikahai	Suelo profundo (deep soil)
*Bomuhai*	‘Bomuhai	Suelo arenoso (sandy soil)
*Pehai*	Pěhai	Suelo arcilloso (clayey soil)
*Xinahai*	Xínahai	Suelo somero (shallow soil)
*Xidohai*	Xídóhai	Suelo tepetatoso (tepetate soil)
*T’axahai*	T’ǎxhai, t’axhai	Suelo blanquizco (whitish soil)
*Bospihai*	‘Bospihai	Suelo cenizo (ashen soil)
*Detha*	De̱thä	Maíz (maize)
*Ju*	Jǔ̱	Frijol (beans)
*Guruju*	Guruju̱	Arvejón (peas)
*Uada*	Uä’da	Maguey (maguey)
*Daju*	Däju̱	Haba (fava beans)

Source: local experts from Huitexcalco de Morelos

**Fig. 4:**
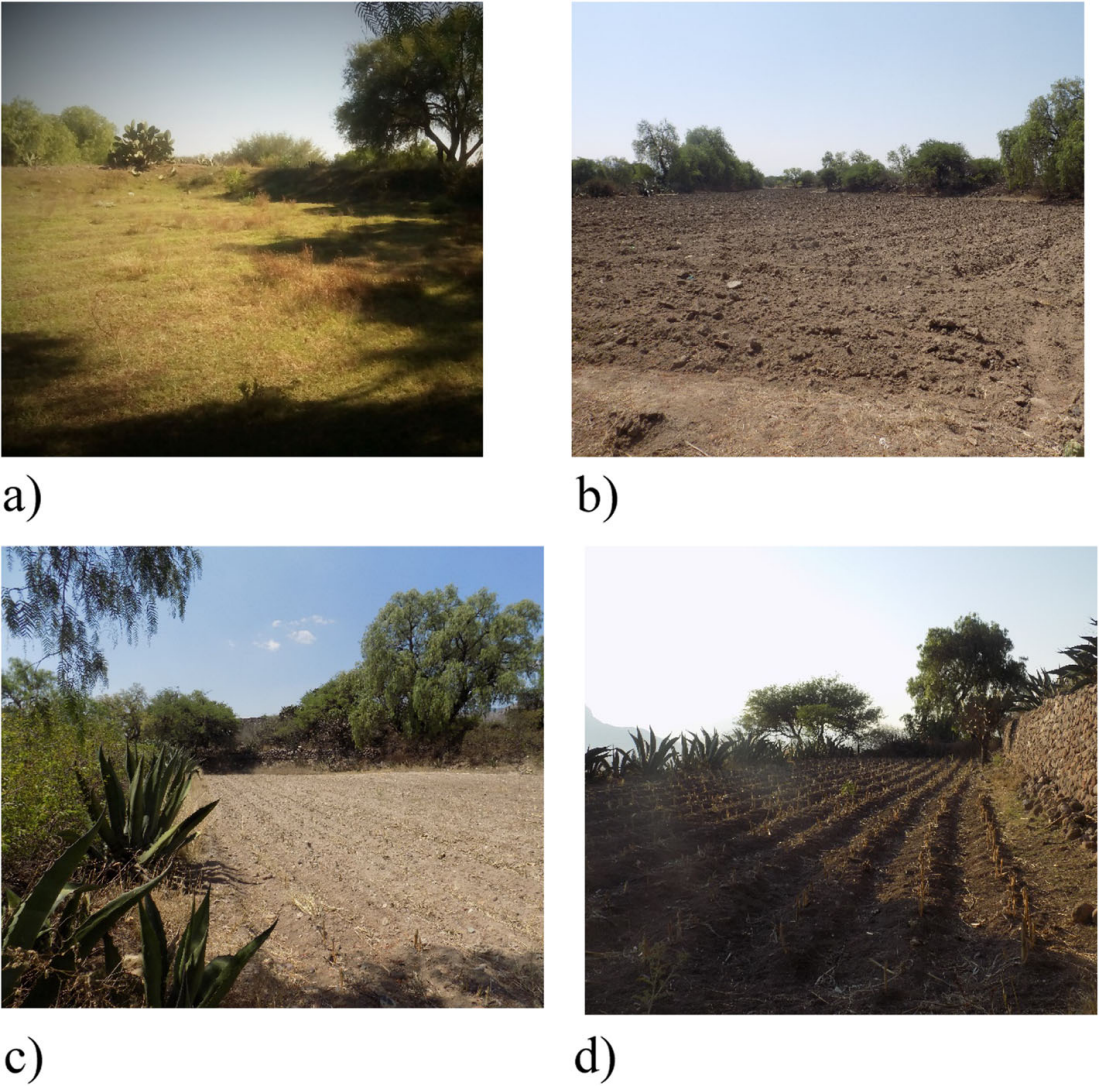
Categories of Hñahñu farmland parcels in Huitexcalco de Morelos, Mezquital Valley, Mexico. **a** A typical and ancient *mothe muiñhe* parcel located at the mouth of a gully with accumulated sediments of more than 2 meters deep and *jodos* defining its border. **b** The parcels of *mothe ñut’athee* are located on the banks of the rivers from where water flows via *ñuthee njushai* and *ñuthee* for irrigation. **c** *Mothe gadñhe* is a variant of the *mothe ñut’athee*. Here, the water returns to the channel at some point where the slope is not favorable. **d** *Mothee* terraces are built to increase soil availability and improve moisture retention

**Fig. 5:**
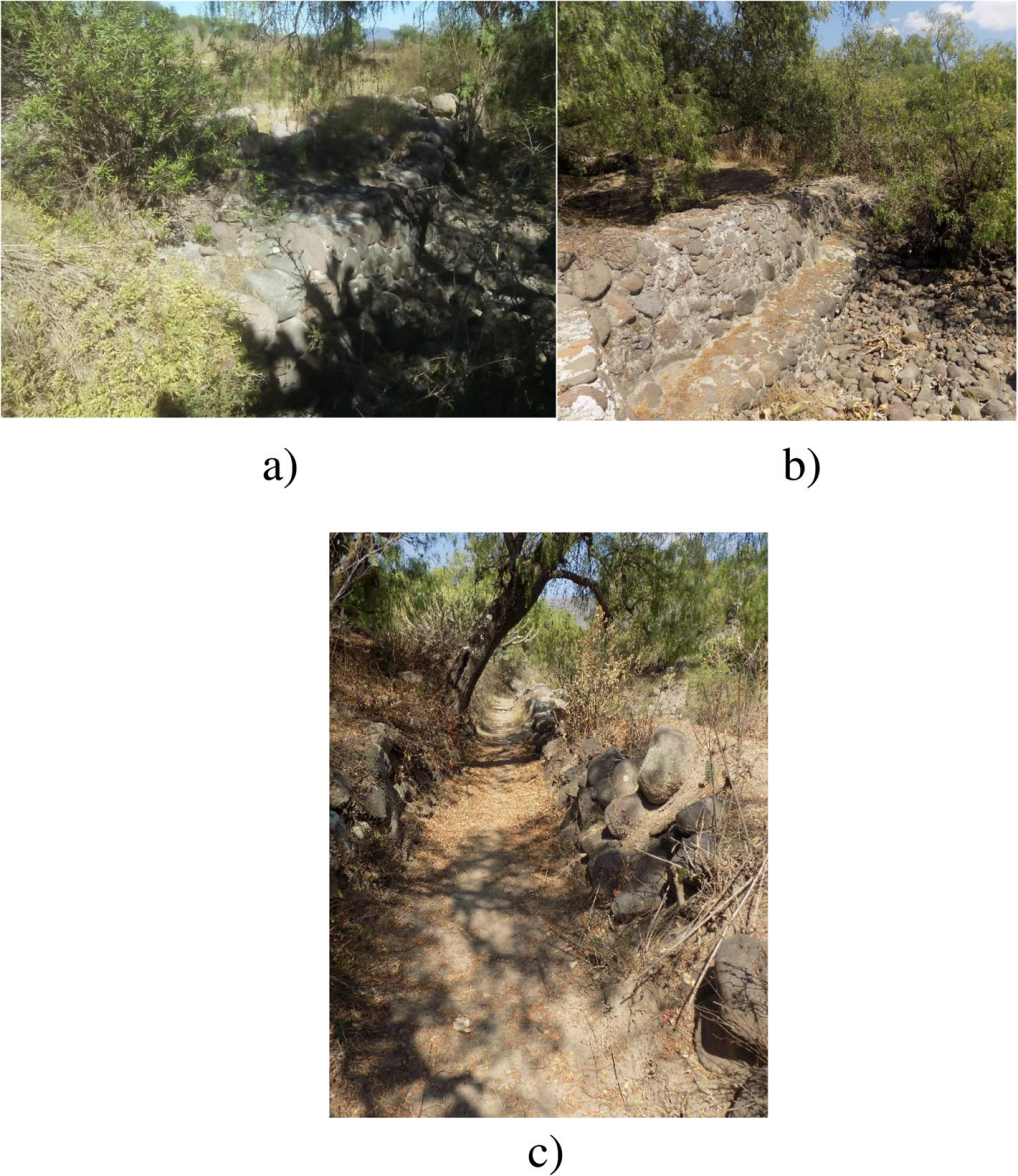
Constitutive components of the Hñahñu farmland parcels in Huitexcalco de Morelos, Mezquital Valley, Mexico. **a** A *jodo* built on a *mothe muiñhe* to retain water and favor the accumulation of sediments. **b** The *ndosthee* temporarily retain water which then flows through the channels towards the parcels of *mothe gadñhe*. **c** The *ñuthee njushai* allow water to be carried to farming parcels of *mothe ñut’athee* farther away from the bed of a stream

Farmland parcels that are classified as *mothe muiñhe* are situated on *bobatha*, more precisely—at the mouth of a gully (Table 2; Fig. 4a). More than any other type of farmland, *mothe muiñhe* reflects the extensive experiences of Hñahñu farmers in integrating their ecological knowledge into their cropping. Farmland parcels of *mothe muiñhe* are constructed in a careful manner. First, the shallowest and narrowest gullies in *bobatha* are chosen to make sure that water is retained with minimal effort. Second, *jodos* (stone barriers; Table 2; Fig. 5a) are raised high enough (2.5 m in height and 10 m in length) to retain water for as long as it is possible. *Jodos* are equipped with spillways that allow excessive water to be released. Whether temporal or permanent, *jodos* are annually rebuilt during the dry season in order to capture as much water as possible. Nutrient-rich sediments are accumulated behind *jodos*. *Picahai* (“deep soils”)—a dark sediment (*‘bohai*) sandy texture soil (*bomuhai*)—is considered to be the most fertile for agriculture [37]. A *mothe muiñhe* parcel is completed when a *jodo*-like stone wall is raised on its margins with living barriers of agave plants (*uada*) and fruit trees to reinforce the barrier structure and to complement family diet and income [38]. *Mothe muiñhe* becomes a true soil and moisture containers where maize (*detha*) is successfully grown along with both pole beans (*bat´aju*) and fruit trees.

Hñahñus tend to associate *mothe ñut’athee* parcels with the banks of gullies occurring on *bobatha* and the *nga’ti* of hillsides and low hills (Table 2; Fig. 4b). Slope steepness is imperative to designate these types of lands; only those with enough slope to channel water flows are included in this category. Stone barriers known as *ndosthee ga do* (Table 2; Fig. 5b) are paramount in this operation; they can be as high as 1 m at the mouth of a gully, and their purpose is not to store water, but rather to enable water flows. Water is transported to the parcels through channels referred to as *ñuthee njushai* (Table 2; Fig. 5c) dug in the ground at the same level as *ndosthee ga do*.

Hñahñus are also able to divert water to parcels situated further away from gullies using a system of simple channels known as *ñuthee*, which are dug directly into the ground. Most of the times, retained water is enough to irrigate these parcels. However, if the slope is not favorable or channels are insufficient somewhere along its path, water goes back to the gully. Hñahñus use the term *gadñhe* (Table 2; Fig. 4c) to designate these particular parcels and express how water “flows around” the parcel and returns to the gully through *sokdehe* spillways. Regardless of the differences between *mothe ñut’athee* and its variant (*mothe gadñhe*), these parcels produce good yields of maize and *mu* (squash) alternated with beans, *daju* (fava beans), and *guruju* (peas). This is partly explained by the formation of *picahai*, a form of water-carried soil that is imbued with copious amounts of plant organic matter (*mastee*), which are locally described as “fertile and deep.” Sediments of *picahai* are primarily silty, although sandy (*bomuhai*) and clayey (*pehai*) *picahai* can also be found. Similar to the previous category (*mothe muiñhe*), barriers are raised at the parcel margins and then reinforced with maguey plants.

*Mothe mothee* are established on the *donts’i* of hillsides and low hills where the lack of surface water leads to rainwater collection (Table 2; Fig. 4d). Here, soils are shallow (*xinahai*) with high tepetate (*xidohai*) content and reduced organic matter, which is reflected in their whitish (*t’axahai*) and ashen (*bospihai*) colors. Soil and water management strategies have been implemented in order to overcome these challenges. Hñahñu farmers raise stones walls or *jodos* at the ground level in order to increase the extension of arable areas and soil depth, and retain a larger amount of rainwater. *Jodos* can be as high as 1 m. The edges of terraces are usually planted with *uada* (maguey or agave) in order to reinforce the structure. The process is time consuming, but it certainly leads to soil accumulation, organic fertilization, and intercropping of maize/beans/*guruju* (peas) showing considerable success after just a few years.

### Mapping the Hñahñu farmland system categories

**Fig. 6:**
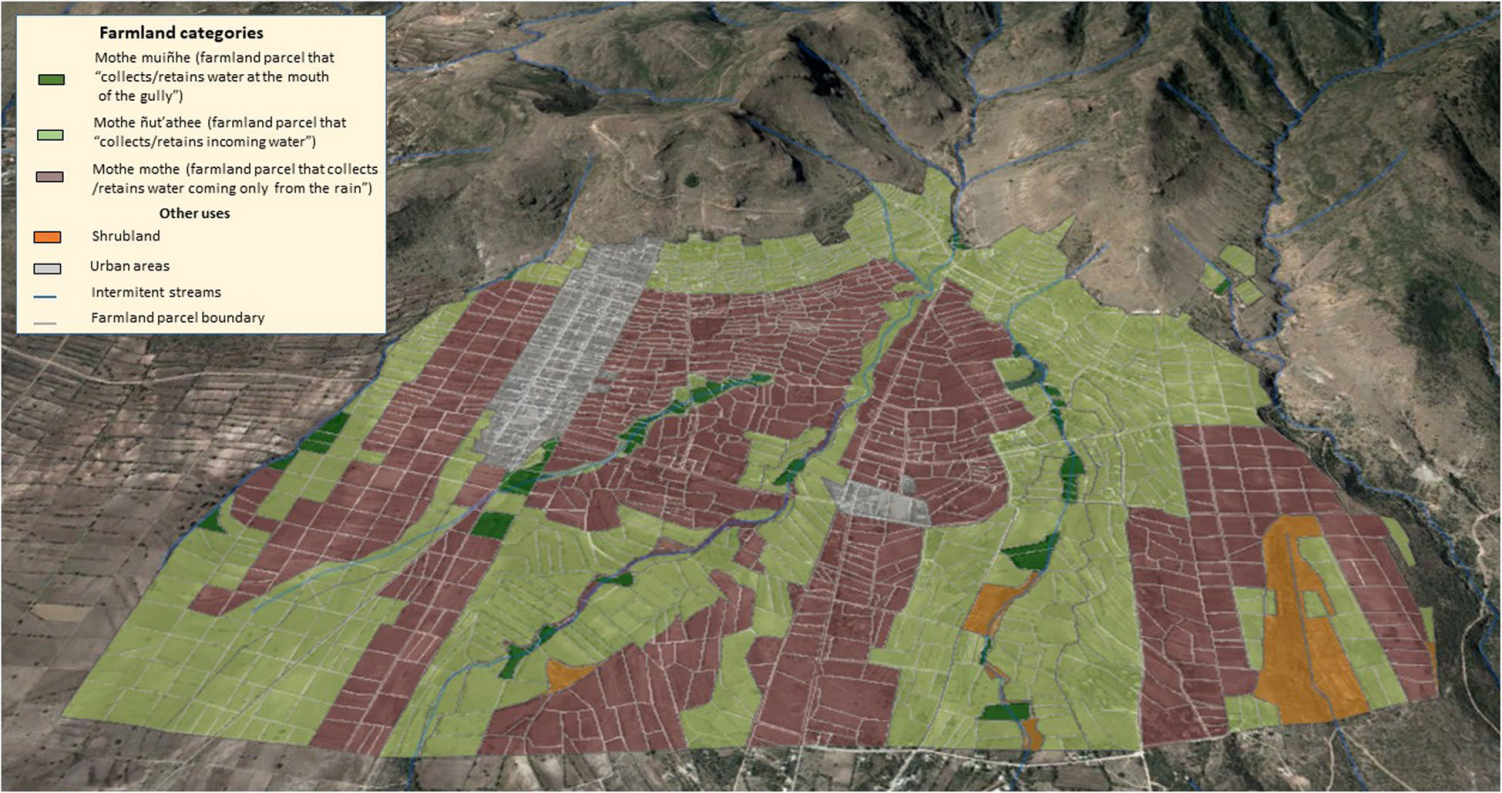
3D map of the Hñahñu farmland categories of Huitexcalco de Morelos, Mezquital Valley, Mexico

Figure 6 summarizes the collective effort of workshop participants attempting to identify the semantic categories and their relationship with the local landscape. At least four aspects can be mentioned concerning this relationship. First, all categories expressed in Hñahñu can be clearly identified in the current landscape. Due to the small numbers of *gadñhe*, we categorize them together with *mothe ñut’athee*.

Second, *mothe muiñhe* polygons largely coincide with the gullies occurring on *bobatha* (plains). Parcels of *mothe muiñhe* occupy a very small area (2.55%) of the map, probably because few gullies in Huitexcalco are able to meet the necessary conditions for establishing a farmland parcel, or because the human labor required to construct the infrastructure that such lands need is not available*.*

Third, although the location of some polygons of *mothe ñut’athee* did coincide with banks on either side of the gullies, this was not a compelling enough reason to identify all parcels of this category in the map. Polygons of *mothe ñut’athee* were observed across large areas of the hillsides—in the *ngats’i* (middle part) and *nga’ti* (lower part) in the eastern side of the community, as well as on low hills to the west. Since a central criterion for recognizing *mothe ñut’athee* was whether or not the parcel had access to irrigation water, the Hñahñu notions of how slope affects the movement of water are necessary. This is the reason why several parcels located next to a gully, but limited by slope, were excluded from this category by the participants.

Finally, it was found that the location of *mothe mothee* coincided with that of the areas which Hñahñu farmers deemed to have lesser agricultural potential. These lands occupied over 43.3% of all the arable area and can be easily distinguished from other land categories of higher agricultural value.

## Discussion

### Taxonomy and drivers of the Hñahñu farmland categories

The organization of Hñahñu farmlands into two levels and four categories is similar to indigenous soil and landscape classifications in many other places, except for the number of categories which is significantly lower here. For example, Mayan farmers’ soil taxonomy includes two levels and seven types, while the Takana people of the Bolivian Amazon classify their landscape into 30 categories based on the rainforest’s successional stages [39, 40]. Differences can be attributed to the characteristics of the ecosystem. The Mezquital Valley has a semi-arid environment, whereas tropical climates in southern Mexico and in the Amazon produce ecologically rich habitats. The Hñahñu farmland system studied here focused on land types rather than on soils whose categories are much more numerous.

Similar categories to those recognized by Hñahñu farmers have been reported for the Chontales, Mayas [39, 41], and the Purepechas [11]. Purepecha farmers classify the relief on the basis of topographic position, as “up” (high), “intermediate” (middle), and “down” (low). In addition, they use criteria including aspect, slope, surface lithology, and adjacency to other relief types, in conjunction with anthropomorphic terms such as head, breast, and foot, to describe multiple configurations. The Csango people in Romania are known to distinguish more categories in valleys than in mountains [42]. The differences with our study can be attributed to the fact that Hñahñu farmers have little arable land and make an optimal use of hillsides and low hills.

Primary drivers used by Hñahñu farmers for distinguishing land types are both physical and functional, i.e., the topographic position of the farmland and the management practices required to obtain irrigation water. Other drivers including soil texture, color, depth, slope variations, and the human resources used to maintain or rehabilitate the infrastructures also significantly influence the designation. These drivers are similar to other local taxonomies; the designation of the soil-ground characteristics is based both on relevant physical properties, such as color and texture, and the productive use [40, 43–45].

### Lexicalization

The majority of terms refer to water or landscape management practices. Several of these terms were topological: *donts’i*, *ngats’i*, and *nga’ti* designate hillside sections on the basis of their relative position, whereas *gadñhe* (“on the banks of the gully”) and *muiñhe* (“at the mouth of the gully”) are positional or locative terms that indicate the part of gully (*hñe*) in which the water is retained. In contrast, the terms *ñut’athee* (“that collects/retains incoming water from guillies or runoff”) and *mothee* (“that collects/retains water coming only from rainfall”) are functional; they elucidate the manner in which water is supplied to these parcels—from the gullies or runoff in the first case, and directly from the terrain in the second case.

Interestingly, participants did not include Spanish terms in the recorded list. These results are different from Takana farmers’ ethnophysiography where Spanish terms prevailed when describing landforms [40]. The abundance of Hñahñu terms identified in this study shows people’s keenness to speak their own language. The composition of our research team also allowed for this to happen, since one of the coauthors of this paper is totally fluent in Hñahñu and a member of the community. This shows how important it is to involve local people in landscape recognition research.

### Mapping the Hñahñu landscape terms

Although different landforms were identified, their boundaries were not. This made the mapping process very difficult. Fortunately, the land categories were properly mapped, so the terms documented in this paper have a factual representation in the local landscape. Similar studies have experienced difficulties in defining the spatial delimitation of local terms, particularly when they are semantically vague and spatially inaccurate [11, 42]. In this study, such a lack of precision was partly overcome because the map did include parcel boundaries. Additionally, a classification rule premised on the predominant type was implemented in order to designate the category when there were several farmland types coexisting in the same parcel. However, vagueness remained in the topographic changes from plains to low hills or hillsides, because participants did not recognize a distinction between them. For the majority of native people, boundaries of large landscape units are often fuzzy and will only be marked definitively through externally induced human demarcation [45–48].

Natural language concerning places and space tends to vary in the degree of recognition and validity by community members [15]. However, the findings suggest that the Hñahñu farming landscape system is common to workshop participants. The terms used to describe their landscape are also valid in their socio-productive space. Management forms are embedded into the terms and are central to providing a definitive designation of the farming parcels.

## Conclusions

This paper sheds light on the relationship between native language and landscape organization among Hñahñu farmers of the Mezquital Valley, Mexico. The paper makes three important contributions. First, it highlights the criteria used for taxonomic organization and the role of scale of perception on spatial properties. Second, it illuminates the semantic structure of native terms used to describe the Hñahñu agricultural landscape. The core components of this structure are topography and access to flood irrigation. Third, although cultural forms of land management are secondary in the process of landscape designation, they are all embedded in each Hñahñu term.

The study takes a step towards the digital representation of agricultural knowledge systems. However, more research is needed on the integration of local knowledge into GIS using participatory mapping techniques that are sensitive to indigenous environments. More attention should be paid to traditional agricultural systems both for their cultural relevance and for their conceptual and practical contributions to sustainable food systems in such places [49].

## Data Availability

All data generated or analyzed during this study are included in this published article (and its supplementary information files).
